# A vast genomic deletion in the C56BL/6 genome affects different genes within the *Ifi200* cluster on chromosome 1 and mediates obesity and insulin resistance

**DOI:** 10.1186/s12864-017-3552-6

**Published:** 2017-02-15

**Authors:** Heike Vogel, Markus Jähnert, Mandy Stadion, Daniela Matzke, Stephan Scherneck, Annette Schürmann

**Affiliations:** 1 Department of Experimental Diabetology, German Institute of Human Nutrition Potsdam-Rehbruecke, Arthur-Scheunert Allee 114-116, D-14558 Nuthetal, Germany; 2 German Center for Diabetes Research (DZD), Ingolstädter Landstr. 1, 85764 München-Neuherberg, Germany; 3 Institute of Pharmacology and Toxicology, University of Braunschweig, Mendelssohnstr. 1, 38106 Braunschweig, Germany

**Keywords:** BAC library, Gene cluster, Deletion, Obesity

## Abstract

**Background:**

Obesity, the excessive accumulation of body fat, is a highly heritable and genetically heterogeneous disorder. The complex, polygenic basis for the disease consisting of a network of different gene variants is still not completely known.

**Results:**

In the current study we generated a BAC library of the obese-prone NZO strain to clarify the genomic alteration within the gene cluster *Ifi200* on chr.1 including *Ifi202b*, an obesity gene that is in contrast to NZO not expressed in the lean B6 mouse. With the PacBio sequencing data of NZO BAC clones we identified a deletion spanning approximately 261.8 kb in the B6 reference genome. The deletion affects different members of the *Ifi200* gene family which also includes the original first exon and 5′-regulatory parts of the *Ifi202b* gene and suggests to be the relevant cause of its expression deficiency in B6. In addition, the generation and characterization of congenic mice carrying the critical fragment on the B6 background demonstrate its crucial role for obesity and insulin resistance.

**Conclusions:**

Our data reveal the reconstruction of a complex genomic region on mouse chr.1 resulting from deletions and duplications of *Ifi200* genes and suggest to be relevant for the development of obesity. The results further demonstrate the complexity of the disease and highlight the importance for studying rare genetic variants as they can be causal for large effects.

## Background

Obesity is the consequence of an imbalance between food intake and energy expenditure resulting in an excess accumulation of body fat. Progress and course of obesity and its associated diseases are dependent on nutritional conditions and on other lifestyle parameters (e.g. physical activity). However, its main basis is the complex, polygenic predisposition consisting of a network of variant genes which are still not completely known and difficult to identify in humans. Genome-wide association studies (GWAs) are commonly used for the identification of disease genes. Nevertheless, the identified loci accounting only for a small proportion of the heritability of a complex disease like obesity [1]. Genomic structural variants (GSVs) may explain rare variants with large effects, which are not readily identifiable via SNP-based methods [2–4].

Genomic linkage studies in rodents are a suitable approach to identify and to study such chromosomal alterations. In a previous study we reported the identification of a major obesity QTL (*Nob3*) on distal mouse chr.1 in an outcross population of the New Zealand obese (NZO) strain, a polygenic mouse model for obesity, and the lean C57BL/6J (B6) mouse. By generating recombinant congenic lines and expression studies we finally identified the *Ifi202b* (*Interferon inducible gene 202b*) gene as the causal variant of the *Nob3* locus. The transcriptional regulator *Ifi202b* is a member of the *Ifi200* gene family, which has also been annotated as the PYHIN family, acknowledging the defining features of an N-terminal pyrin domain and C-terminal HIN domain [5]. The proteins are involved in the defense against infection through recognition of foreign DNA, whereas *Ifi202b* was also shown to be involved in the development of obesity [6]. The gene family is arranged as a cluster on mouse chromosome 1 (1q band H3) between the *Cell adhesion molecule 3* (*Cadm3*) gene and a cluster of olfactory receptors. The *Ifi202b* gene is expressed in various tissues of the NZO strain but not transcribed in B6 mice and we hypothesized that this is due to a deletion of the first exon and the 5′-regulatory region [6]. The lack of *Ifi202b* is specific for C57BL mice (e.g. C57BL/10J, C57BL/6J, C57BLKS/J, and C57BR/sdJ), whereas most other strains (e.g. SJL/Bm, DBA2/J, BALB/cJ, C3H/HeJ, and FVB/NJ) express this gene [7].

In the current study we clarified the exact genomic structural variation causing the *Ifi202b* deficiency and demonstrated that a rare genomic alteration on mouse chr.1 is responsible for the development of obesity. We generated a NZO BAC library and performed a *de novo* assembly of the complex Ifi200 region on mouse chr. 1 by using PacBio long reads, a third generation sequencing (TGS) approach and characterized mice with the affected region in respect to different metabolic traits.

## Methods

### Bacterial artificial chromosome (BAC) library construction and screening

NZO (NZO/HIBomDife) BAC library was constructed from high molecular weight (HMW) genomic DNA processed at Amplicon Express Inc. (Pullman, WA, USA) from liver tissue. All animal experiments were approved by the ethics committee of the State Office of Environment, Health and Consumer Protection (V3-2347-21-2012, Federal State of Brandenburg, Germany). With the restriction enzyme HindIII the HMW DNA was partially digested (average size 135 kb) and ligated into the pCC1BAC vector. Ligations were transformed into DH10B *E.coli* cells and plated on LB agar. Clones were picked and arranged onto 384-well plates, replicated and frozen at −80°C. Screening of the BAC library was also processed by Amplicon Express Inc. by using nylon filters with arrayed library clones (18,432 clones) and digoxigenin (DIG)-labeled probes representing position 11,239–11,453 in the genomic sequence of *Ifi202b* (NC_000067). The DIG-labeled probe was generated from gDNA by PCR using the primers *Ifi202b*_for: TCTTCAGAGTGATGGTGTTCG and *Ifi202b*_rev: TGTTTGCAAGTGAAGATCACAA. The *Ifi202b* probe was found to hybridize to 14 BAC clones with a size of 90–196 kb. Two positive clones with a size of 147 kb and 196 kb were selected for sequencing. Isolation of the high molecular weight plasmid from the *E.coli* cultures was performed with the PhasePrep^TM^ BAC DNA Kit (Micro Scale Preparation, Sigma-Aldrich, Steinheim, Germany) and the BACMAX™ DNA Purification Kit (Biozym, Hessisch Oldendorf, Germany) according to the manufacturer’s instructions. The PhasePrep BAC DNA Kit was used for cell harvesting, lysis, neutralization, and nucleic acid precipitation, whereas digestion of the residual RNA, removal of residual impurities and final precipitation was done with the BACMAX Kit.

### BAC sequencing and sequence assembly

Sequencing of the two BAC clones (mixture, ratio 1:1) and assembling was processed by GATC Biotech AG (Konstanz, Germany) using the SMRT® Technology PacBio RS II. *De novo* assembly of BAC inserts was performed with the standard SMRT Portal Software including quality filtering of the reads, improvement of long reads through alignment of short reads, assembly of long reads, and assembly correction. The assembly of the reads was based on the hierarchical genome-assembly process (HGAP).

### Comparative genomic hybridization assay

Genomic DNA was prepared from the tail of C57BL/6J and NZO/HIBomDife mice. Unamplified genomic DNA was labeled with Cy3 (NZO) or Cy5 (reference strain, C57BL/6J) and hybridization was performed by imaGenes (Berlin, Germany) using the NimbleGen platform.

### Animals

#### Breeding and genotyping

All animal experiments were approved by the ethics committee of the State Office of Environment, Health and Consumer Protection (Federal State of Brandenburg, Germany). NZO mice from our own colony (NZO/HIBomDife) and C57BL/6J (Charles River, Sulzfeld, Germany) were used throughout the study. Mice were kept at a temperature of 20 ± 2 °C with a 12:12 h light-dark cycle and had *ad libitum* access to drinking water and to a high-fat diet (HFD) containing 45 kcal% from fat, 35 kcal% from carbohydrates, and 20 kcal% from protein (D12451, Research Diets, Inc., New Brunswick, USA). Congenic mice were generated on a B6 background and the offspring was selected in each generation for carrying the fragment 163.5–177.7 Mbp from NZO on chr.1 (*Nob3.14*). Phenotypical characterization of female congenic mice were performed in the F10N8 generation. For genotyping, DNA was prepared from mouse tails with a DNA isolation kit based on a salt precipitation method (InViTek, Berlin, Germany) and used for tests with polymorphic microsatellite markers. Microsatellites (D1Mit143 and D1Mit115) were genotyped by PCR with oligonucleotide primers obtained from MWG (Ebersberg, Germany), and the microsatellite length was determined by non-denaturing polyacrylamide gel electrophoresis.

#### Body composition and blood glucose

Fat mass of *Nob3.14* mice were determined by nuclear magnetic resonance (EchoMRI™-100H, EchoMRI LCC, Houston, USA) and blood glucose levels were measured in the morning (7–10 a.m.) using a CONTOUR® XT glucometer (Bayer, Leverkusen, Germany).

#### Histological analysis of adipose tissue

Paraffin sections (2 μm) of gonadal white adipose tissue (gonWAT) of 30-week-old *Nob3.14* mice were stained with hematoxylin and eosin. Microscopic images were captured with the Keyence BZ-9000 fluorescent microscope and the corresponding BZ-II Analyzer software (Keyence International, Mechelen, Belgium).

#### Metabolic phenotyping

Oral glucose tolerance tests (OGTT) were performed in 22-week-old mice. Mice were fasted overnight and received 2 g/kg body weight of glucose (Glucosteril® 20%, Fresenius Kabi, Bad Homburg, Germany). Blood glucose and insulin concentrations were detected up to 120 min.

#### Plasma analysis

Plasma insulin levels were analyzed using the Mouse Ultrasensitive Insulin ELISA (ALPCO Diagnostics, Salem, USA) following the manufacturer’s instructions.

#### Protein extraction and western blotting

Adipose tissue of *Nob3.14* mice were homogenized in TES buffer (20 mM TrisHCl, 1 mM EDTA, 8.7% sucrose, pH 7.4, supplemented with protease inhibitor cocktail). Proteins were separated by SDS-PAGE, transferred to a PVDF membrane (Immobilon-P Membrane, Merck Milipore, Darmstadt, Germany) and targeted proteins were detected by ECL Prime Detection Reagent (GE Healthcare Europe GmbH, Freiburg, Germany) using the FUSION-SL4 advanced chemiluminescence system (Peqlab Biotechnologie GmbH, Erlangen, Germany). Primary antibodies against PPARγ (ab41928, Abcam, Cambridge, UK), pHSL (#4139S, Cell Signaling, Beverly, MA, USA), tHSL (#4107S, Cell Signaling), β- ACTIN (A3854, Sigma-Aldrich, St. Louis, USA), and appropriate horseradish peroxidase-labeled secondary antibodies (Dianova, Hamburg, Germany) were applied.

## Results and discussion

The *Ifi200* gene cluster developed as a consequence of gene duplications and rearrangements resulting in a divergence in the number of genes between various inbred strains of mice and in repetitive sequences even in coding regions between the different gene members. In order to clarify the genomic alteration responsible for the *Ifi202b* deficiency in the B6 mouse we used the PacBio system, single-molecule real-time (SMRT) sequencing approach, for *de novo* assembling of the critical region in the NZO strain.

**Fig. 1:**
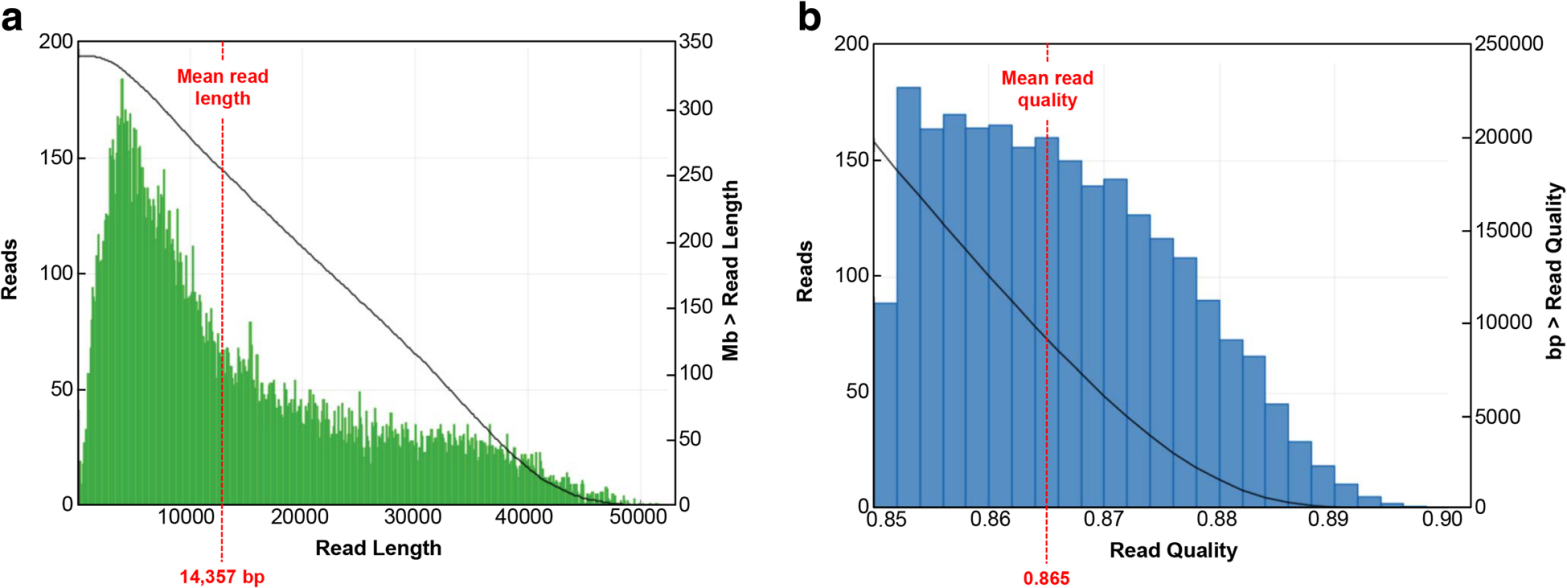
PacBio sequencing parameters. **a** Read length distribution of the 17,802 PacBio reads with an average read length of 14,357 bp and maximal read length of 30,378 bp (after quality trimming). **b** Quality distribution of the PacBio reads with an average quality of 0.865

**Fig. 2:**
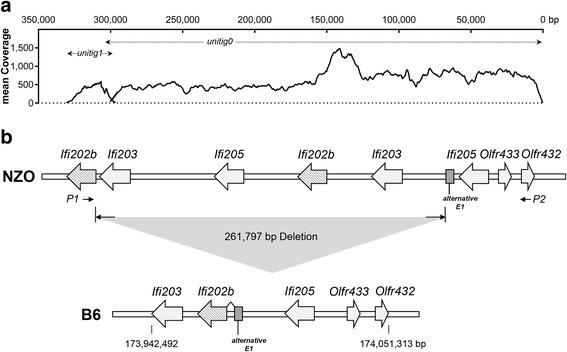
Identification of a B6-specific deletion in the *Ifi200* gene cluster. **a** Observed depth of coverage across unitig0 and unitig1 after *de novo* assembly of the PacBio reads. **b** Schematic overview of the *de novo* assembly results representing genes within the *Ifi200* gene cluster. A direct comparison of the genomic NZO sequence with the B6 reference genome revealed a 261,797 bp deletion including copies of the Ifi200-family members, *Ifi203*, *Ifi205*, the first exon and the 5′-regulatory part of the *Ifi202b* gene. As consequence, an intronic sequence (alternative E1) in NZO is spliced to exon 2 of *Ifi202b* in the B6 genome. P1 and P2: probes used for the screening of NZO BAC clones containing the *Ifi202b* region on chr.1

For the screening of the NZO BAC clones containing the relevant *Ifi202b* upstream sequence a probe matching a unique *Ifi202b* sequence was used. Additionally a probe specific for the *Olfr432* gene was chosen to define the distal border of the region of interest; in contrast to the genomic *Ifi200* region the *Olfr432* gene represents a unique sequence within the mouse genome. In total, sequencing of the NZO BAC clones mapped 17,802 PacBio RS reads with a mean read length of 14,357 kb (maximal read length 30,378 kb) and a mean read quality of 0.865. *De novo* assembly of the reads resulted in 4 contigs. However, two of them were not considered for further analysis (unitig2: 35 kb, mean coverage 24 and unitig3: 38 kb, mean coverage 26) due to poor sequence quality. With the two remaining contigs (unitig1: 36.5 kb, mean coverage 365 and unitig0: 300 kb, mean coverage 603; Fig. 1a and b) it was possible to assemble a region covering 6 genes that belongs to the *Ifi200* gene family and the olfactory receptor *Olfr433* as the distal boundary (Fig. 2b, upper panel). As described earlier the NZO strain carries two copies of the *Ifi202b* gene which differ in only 8 bp within the coding region, respectively 7 amino acids [6]. In addition, sequence analysis of the BAC identified two copies of other family members; *Ifi205* and *Ifi203*. Interestingly, by comparing the assembled NZO sequence with the B6 reference genome we identified a 261,797 bp deletion affecting the *Ifi200* locus in respect to gene duplications.

With a second-generation sequencing (SGS) approach it would have been impossible to solve the organization of the *Ifi200* cluster in NZO as sequences are mapped to the B6 reference genome and gaps within the reference genome will result in an incorrect alignment [8]. While the SGS approach is efficient for accurately identifying SNPs in the genome, it does not enable a thorough characterization of structural variations such as insertions and deletions [9–11]. The short sequence read data has complicated the assembly of repetitive structures leading to the translation into gaps, missing data and more incomplete assembly [12–14]. In contrast, the main advantage of TGS is the long read nature, which was reported to be as long as 3,000 bp on average, and some reads are supposed to be 20,000 bp or even longer. The long read length provides an important benefit for *de novo* assemblies, it allows the discovery of large structural variants, and it provides accurate microsatellite lengths, detection of sensitive SNPs, and haplotype blocks [8, 15–18]. TGS has successfully been used for *de novo* assembling of hundreds of microbial genomes and reconstruction of plant and animal genomes [18–23]. It has also been applied to resequencing analysis, to create detailed maps of structural variations and phasing variants across large regions of human chromosomes [23–25].

**Fig. 3:**
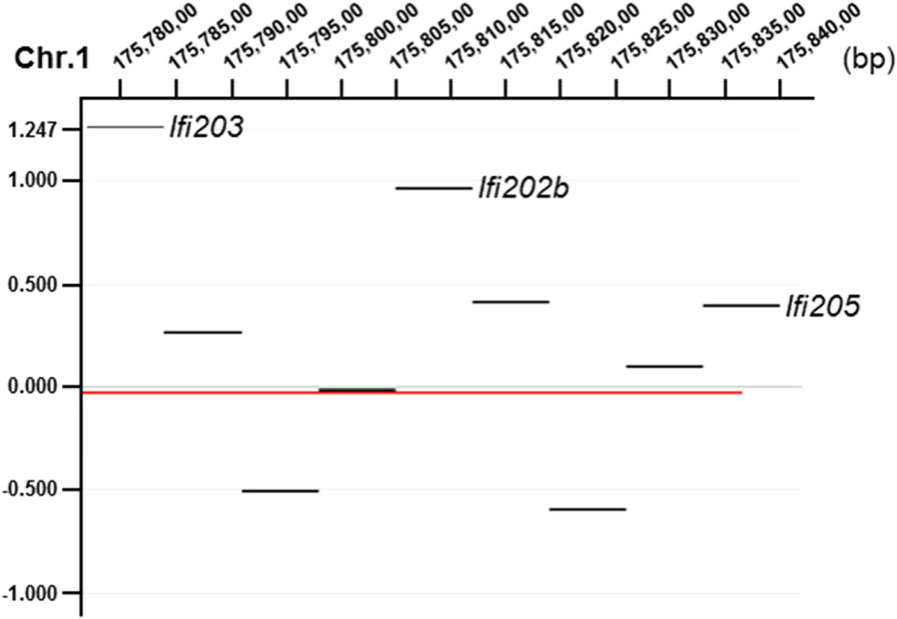
Identification of copy number variations (CNVs) within the *Ifi200* cluster. Results obtained from a comparative genomic hybridization assay (CGH) of genomic DNA from the B6 and NZO strain (NCBI Build 36, mm8). Shown are the positions of the critical Ifi200 cluster. The red line represents equal copies in B6 and NZO, whereas areas above 0.0 indicates that two or more copies exist in NZO. Regions corresponding to *Ifi203*, *Ifi202b*, and *Ifi205* are highlighted

The evolutionary analysis revealed a remarkable plasticity in the mammalian *Ifi200* genes, suggesting the existence of strong evolutionary pressures that have shaped the *Ifi200* sequences and functions throughout the mammalian lineage [26]. Here, we report the identification of structural variations within the *Ifi200* (*PYHIN*) gene cluster in the obese NZO strain. Cridland and colleagues presented a map comparing the human, C57BL/6 mouse, and rat *Ifi200* gene loci. The mouse contains at least 14 mouse *Ifi200* genes, whereas the human and rat genome expresses only 4, respectively 5 [5]. It was already published that the *Ifi200* gene locus is divergent between various mouse strains as the number of genes present at the locus and the sequence is different [5, 6]. The number of predicted mouse genes has increased with each new update of the mouse genome database and in the current study with *de novo* assembling of the PacBio sequencing reads we can strengthen and expand this assumption to the obese NZO strain [5]. The NZO strain carries two copies of *Ifi202b* (*Ifi202a and b*) which was also found in the 129X1/SvJ mouse genome in addition to a pseudogene (*Ifi202c*), whereas only one truncated copy is present in C57BL/6 that is not expressed in metabolically relevant tissues [6, 27, 28]. Another family member, *Ifi203*, showed two extra copies in NZO in comparison to B6. Also the *Ifi205* gene was duplicated as two regions, spanning the coding sequence of the gene, could be mapped in the NZO BAC clones (Fig. 2b). To further verify the sequencing results we performed a comparative genomic hybridization assay (CGH) of genomic DNA obtained from the B6 and the NZO strain to detect copy number variations (CNVs) within the cluster. This analysis further supports that the NZO strain carries at least two copies of the genes *Ifi202b*, *Ifi203*, and *Ifi205* (Fig. 3). Other studies also show the presence of gene duplications. She and colleagues (2008) assessed CNVs between the B6 strain and 15 mouse strains (including NZO) which were used for genetic association studies, sequencing, and the Mouse Phenome Project [29]. The analysis also showed a duplication of the *Ifi203* gene. Similar results were detected for *Ifi205* in the study by Cahan et al., 2009 where CNVs in 17 mouse strains were analyzed [30]. In conclusion, *de novo* assembling of the NZO BAC clone reads and the analysis of CNVs revealed structural variations between different inbred strains of mice within a complex region on chr. 1 caused by duplications and genomic alterations.

It is also documented that the corresponding region in humans is affected by genomic alterations. According to the 1000 Genomes project several deletions, CNV , and duplications can be mapped within this locus [31]. Cagliani and colleagues performed an evolutionary analysis of the human family members (*MNDA*, *PYHIN1*, *IFI16*, and *AIM2*) by analyzing inter- and intraspecies diversity and revealed that the genes have been repeatedly targeted by natural selection. Especially the *IFI16* gene region shows a high nucleotide diversity in human populations and indicates that the region has been a target of long-standing balancing selection [32].

The main goal of the current study was to analyze the chromosomal alterations leading to the *Ifi202b* deficiency in the B6 strain. With the BAC sequencing we identified a deletion spanning approximately 261.8 kb within the B6 genome, a sequence present in NZO. The deletion includes different copies of *Ifi200*-family members, *Ifi203*, *Ifi205*, and exon 1 of *Ifi202b* (Fig. 2b). In our previous study we identified an alternative first exon in the B6 reference genome (Vogel et al., 2012). With the current study we are finally able to define the exact chromosomal region deleted in B6 and we can explain how this alternative exon 1 - which is an intronic sequence in NZO - is spliced to exon 2 of *Ifi202b* in the B6 genome (Fig. 2b, lower panel). The fact that B6 do not express *Ifi202b* in the same tissues (e.g. adipose tissue, liver, and skeletal muscle) as NZO indicates that in addition to the first exon also the promotor or at least part of it was deleted as well.

It is also reasonable to assume that the deleted region in B6 contains enhancer motifs/long-range control elements that drive and regulate the expression of other genes. In a previous study we reported that the genes *Lefty1*, *Pcp4l1*, and *Apoa2*, located in the same diabesity susceptibility locus as *Ifi202b* (*Nob3*), are exclusively present in islets of the diabetes-resistant B6 strain in contrast to the diabetes-prone NZO mouse. The identified genes are furthermore involved in the adaptive islet hyperplasia and prevention from severe diabetes in B6-*ob/ob* mice [33]. With the hereby reported data we hypothesize that the genomic alterations within the cluster may also include enhancer elements that carry the potential to regulate the expression of *Lefty1*, *Pcp4l1*, and *Apoa2*. By using the *Nsite* program, a computer tool to search for regulatory elements (REs), we found 5 predictive enhancer motifs that are located within the deleted sequence in the B6 genome which can potently be responsible for the described expression differences. A number of longe-range regulatory disruptions affecting the expression of genes have already been described [34, 35]. One of the oldest examples of a human gene in which long-range regulations has been implicated and studied is *SOX9*, a gene responsible for autosomal sex reversal and Campomelic Dysplasia (CD). All rearrangements including deletions are found from 50 kb to 950 kb upstream of *SOX9* suggesting that a similar mechanism could also account for the expression differences between the diabetes-prone NZO and diabetes-resistant B6 strain of genes located within the *Nob3* locus [34, 35].

**Fig. 4:**
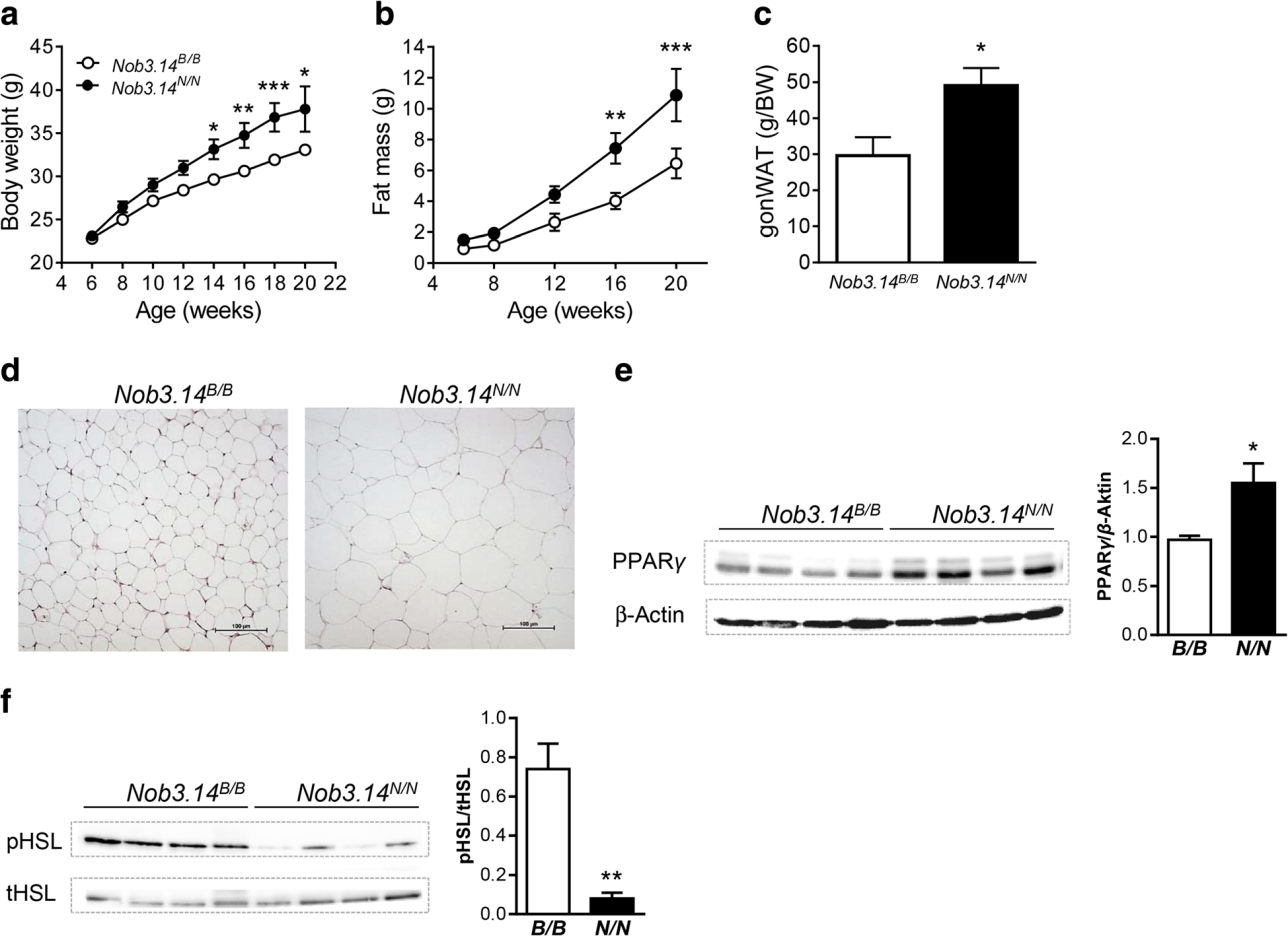
Insertion of the genomic NZO fragment containing the Ifi200 cluster into the B6 strain induces obesity. Body weight (**a**) and fat mass (**b**) development of *Nob3.14* ^*B/B*^ (*n* = 9) and *Nob3.14* ^*N/N*^ (*n* = 9) female mice kept on HFD. **c** Gonadal white adipose tissue (gonWAT) mass of *Nob3.14* female mice (*n* = 6). **d** Histological analysis of gonWAT of *Nob3.14* ^*B/B*^ and *Nob3.14* ^*N/N*^ mice. Scale bar, 50 μm. Western blot analysis indicated an increased expression of the adipogenic marker PPARy (**e**) and the lipolytic enzyme pHSL (**f**) in gonWAT of congenic mice carrying the *Nob3.14* ^*N/N*^ locus in comparison to controls (*Nob3.14* ^*B/B*^). Data are presented as mean ± SEM. *p < 0.05, **p < 0.01, ***p < 0.001 by *t*-test

**Fig. 5:**
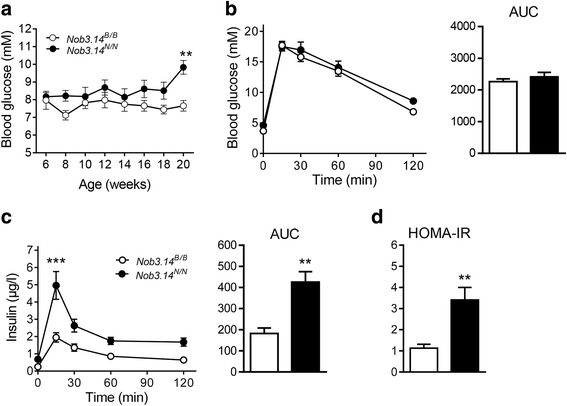
Impaired insulin sensitivity in animals carrying the NZO *Ifi200* gene cluster. **a** Blood glucose levels in *Nob3.14* ^*B/B*^ and *Nob3.14* ^*N/N*^ female mice under HFD conditions. **b** Female congenic mice (*Nob3.14* ^*B/B*^ (*n* = 8) and *Nob3.14* ^*N/N*^(*n* = 10)) were fasted overnight and received an oral bolus of 2 g/kg body weight of glucose and blood glucose (**b**) and insulin levels (**c**) were measured at the indicated time points. **d** Calculation of the HOMA-IR of congenic mice (*Nob3.14* ^*B/B*^, *n* = 6; *Nob3.14* ^*N/N*^, *n* = 9). Data are presented as mean ± SEM. **p < 0.01, ***p < 0.001 by *t*-test

Finally, to elucidate whether the genomic alteration on chr. 1 is also associated with metabolic alterations we generated and characterized congenic mice carrying 14.2 Mbp (163.5-177.7 Mbp) of the NZO genome (*Nob3.14* ^*N/N*^), including the Ifi200 gene cluster, on B6 background. On HFD, homozygous NZO allele carriers developed a higher body weight and fat mass (Fig. 4a and b), in particular gonadal white adipose tissue (gonWAT, Fig. 4c), than the corresponding controls (*Nob3.14* ^*B/B*^). Histological analysis of the gonWAT demonstrated that the adipocytes were larger in the *Nob3.14* ^*N/N*^ group than those of *Nob3.14* ^*B/B*^ mice (Fig. 4d). As these data points towards a role of the cluster in adipose tissue biology we tested the expression of proteins involved in adipocyte differentiation and lipolysis. Western blot analysis indicated an increased expression of the adipogenic marker PPARy (Peroxisome proliferator-activated receptor gamma) and a decreased activation of the lipolytic enzyme HSL (Hormone sensitive lipase) in gonWAT of NZO allele carriers in comparison to controls (Fig. 4e and f). As obesity and hypertrophy of adipose tissue are also known to impair insulin sensitivity and glucose tolerance, we measured the glucose levels of the congenic lines. Blood glucose levels were measured randomly and started to differ at the age of 20 weeks between the two groups with higher concentrations in NZO allele carriers (Fig. 5a). Glucose clearance during oral glucose tolerance tests was not different between the two genotypes (Fig. 5b). However, the *Nob3.14* ^*N/N*^ mice required higher levels of insulin than *Nob3.14* ^*B/B*^ mice to clear blood glucose, pointing towards an insulin resistance (Fig. 5c) which is also indicated by calculating the HOMA-IR (Fig. 5d). In conclusion, introducing the genomic region of the *Ifi200* gene cluster of the NZO genome into the B6 genome results in the development of obesity and is associated with insulin resistance which demonstrates the functional consequences of the alteration on chr.1.

In different reports it was already published that rare GSVs are associated with obesity [36]. A rare (0.7%), 593 kb deletion on chromosome 16p11.2 (at 29.5–30.1 Mbp) was shown to be significantly (*p* = 6.4 × 10^−8^) enriched in obese patients compared to controls, whereas a duplication of the same locus has the opposite effect, being associated with underweight [1, 37, 38]. Another study by Wang et al. [39] also showed large and rare CNVs that are associated with a higher risk to develop obesity. They reported several CNVs that affect known candidate genes for obesity, such as a 3.3-Mbp deletion disrupting *NAP1L5* and a 2.1-Mbp deletion disrupting *UCP1* and *IL15*. One prominent example for chromosomal syndromes with obesity is the Prader-Willi syndrome (PWS) in which a 5–7 Mb deletion of the paternally inherited chromosomal 15q11.2-q13 region is responsible for a neurobehavioral disorder manifested by infantile hypotonia and feeding difficulties in infancy, followed by morbid obesity secondary to hyperphagia [40].

## Conclusions

In summary, by using TGS it was possible to assemble a complex genomic region on mouse chr. 1 containing different genes of the *Ifi200* cluster. This approach further leads to the identification of a vast chromosomal deletion including the regulatory part of the obesity-associated gene *Ifi202b*, as well as one copy of *Ifi203* and one of *Ifi205* in the B6 strain which finally leads to an altered expression and consequently affecting the susceptibility to develop obesity.

## Abbreviations

*AIM2*: *Absent in melanoma 2*
*Apoa2*: *Apolipoprotein A-II*
*B/B*: *B6/B6*
B6: C57BL/6
BAC: Bacterial artificial chromosome
*Cadm3*: *Cell adhesion molecule 3*
CGH: Comparative genomic hybridization
chr.: Chromosome
CNVs: Copy number variations
Cy: Cyanine
DIG: Digoxigenin
*E.coli*: *Escherichia coli*
E1: Exon 1
EDTA: Ethylenediaminetetraacetic acid
ELISA: Enzyme linked immunosorbent assay
for: Forward
gDNA: Genomic DNA
gonWAT: Gonadal white adipose tissue
GSVs: Genomic structural variants
GWAs: Genome-wide association studies
HFD: High-fat diet
HGAP: Hierarchical genome-assembly process
HMW: High molecular weight
HOMA-IR: Homeostasis model assessment of insulin resistance
HSL: Hormone sensitive lipase
*IFI16*: *Interferon gamma inducible protein 16*
*Ifi200*: *Interferon inducible gene 200 family*
*Ifi202b*: *Interferon inducible gene 202b*
*Ifi203*: *Interferon inducible gene 203*
*Ifi205*: *Interferon inducible gene 205*
*IL15*: *Interleukin 15*
LB: Lysogeny broth
*Lefty1*: *Left right determination factor 1*
*MNDA*: *Myeloid cell nuclear differentiation antigen*
*N/N*: *NZO/NZO*
*NAP1L5*: *Nucleosome assembly protein 1 like 5*
*Nob3*: *NZO obesity 3*
NZO: New Zealand obese
OGTT: Oral glucose tolerance test
*Olfr432*: *Olfactory receptor 432*
*Olfr433*: *Olfactory receptor 433*
PacBio: Pacific biosciences
*Pcp4l1*: *Purkinje cell protein 4-like 1*
*PPARγ*: *Peroxisome proliferator-activated receptor gamma*
PVDF: Polyvinylidene difluoride
PWS: Prader-Willi syndrome
PYHIN: Pyrin and HIN domain-containing protein
P1/2: Primer 1/2
QTL: Quantitative trait locus
rev: Reverse
SDS-PAGE: Sodium dodecyl sulfate polyacrylamide gel electrophoresis
SGS: Second-generation sequencing
SMRT: Single-molecule real-time sequencing
SNP: Single nucleotide polymorphism
*SOX9*: *SRY (sex determining region Y)-box 9*
TGS: Third-generation sequencing
*UCP1*: *Uncoupling protein 1*
